# Curcumin/L‐OHP co‐loaded HAP for cGAS‐STING pathway activation to enhance the natural immune response in colorectal cancer

**DOI:** 10.1002/btm2.10610

**Published:** 2023-10-14

**Authors:** Yao Xiao, Guohu Guo, Huaiming Wang, Bin Peng, Yinglin Lin, Gaowen Qu, Ben Li, Zhaojun Jiang, Fan Zhang, Jiaming Wu, Min Liang

**Affiliations:** ^1^ Department of Oncology The Fifth Affiliated Hospital of Guangzhou Medical University, Guangzhou Medical University Guangzhou China; ^2^ The Key Laboratory of Biological Targeting Diagnosis, Therapy and Rehabilitation of Guangdong Higher Education Institutes The Fifth Affiliated Hospital of Guangzhou Medical University, Guangzhou Medical University Guangzhou China; ^3^ Department of Vascular and Gastroenterology Surgery Second Affiliated Hospital of Shantou University Medical College Shantou China; ^4^ Department of Colorectal Surgery, Laboratory of Colorectal and Pelvic Floor Disease The Sixth Affilliated Hospital, Sun Yat‐sen University Guangzhou Guangdong P.R. China; ^5^ Department of Gastrointestinal Surgery The Fifth Affiliated Hospital of Guangzhou Medical University, Guangzhou Medical University Guangzhou China; ^6^ Department of Gastrointestinal Surgery The First Affiliated Hospital of Jiaxing University Jiaxing China

**Keywords:** Ca^2+^ overload, cGAS/STING, innate immunity, mtDNA, nDNA

## Abstract

Insufficient immune cell infiltration into the tumor microenvironment (TME) greatly compromises the clinical application of immune‐checkpoint inhibitors (ICIs)‐based immunotherapy. Recent findings have shown that activation of the cyclic GMP‐AMP synthase‐stimulator of interferon genes (cGAS‐STING) pathway can enhance natural immunity and increase lymphocyte infiltration into the TME, which presents a promising strategy for cancer immunotherapy. In this study, we constructed hydroxyapatite nanoparticles co‐loaded with curcumin and L‐oxaliplatin (Cur/L‐OHP@HAP NPs). We analyzed the particle‐size distribution, zeta potential, spectral characteristics (Fourier‐transform infrared spectroscopy, X‐ray photoelectron spectroscopy, ultraviolet–visible spectroscopy), and drug‐release properties of the Cur/L‐OHP@HAP NPs. The cellular uptake of the Cur/L‐OHP@HAP NPs detected by flow cytometry and confocal laser‐scanning microscopy. We comprehensively evaluated the anti‐tumor properties and immune‐activating effects of the NPs, both in vitro and in vivo. Physicochemical characterizations demonstrated that the Cur/L‐OHP@HAP NPs were successfully synthesized and were capable of pH‐dependent drug release. Notably, the Cur/L‐OHP@HAP NPs efficiently entered cancer cells, after which the released L‐OHP induced nuclear DNA (nDNA) damage to some extent. HAP promoted the release of intracellular Ca^2+^ stores in cancer cells, and curcumin inhibited Ca^2+^ efflux, resulting in intracellular Ca^2+^ overload and the release of mitochondrial DNA (mtDNA). Damage to both nDNA and mtDNA greatly stimulated the cGAS‐STING pathway, thereby activating natural immunity, accompanied by immune cell recruitment to the TME. In summary, the Cur/L‐OHP@HAP NPs show good prospects for improving cancer immunotherapy.

**Translational Impact Statements**

This study achieved effective tumor control by enhancing tumor immune response, and provided potential therapeutic strategies for clinical application.

## 1 INTRODUCTION

Colorectal cancer (CRC) is the third leading cause of cancer‐related deaths worldwide, affecting approximately 1.85 million people annually and resulting in 850,000 deaths.^1^ Owing to the insidious symptoms of CRC at an early stage, approximately 80% of patients in China are diagnosed at an advanced stage when radical surgery is no longer possible.^2^ Patients with advanced‐stage CRC can rely only on chemotherapy‐based, palliative‐treatment programs, and chemoresistance during the treatment process often significantly reduces the therapeutic efficacy.^3^ Therefore, novel treatments with enhanced therapeutic efficacies are urgently needed.

Data published in recent years have shown that immune‐checkpoint inhibitors (ICIs), including programmed cell death 1 (PD‐1)‐inhibitor monotherapy, combination therapy with PD‐1 and cytotoxic T‐lymphocyte associated protein 4 (CTLA4) inhibitors, and combination therapy with a PD‐1 inhibitor and an antibody against vascular endothelial growth factor, can maximize the anti‐tumor effects of T cells by relieving tumor cell‐dependent inhibition of T cell functions.^4^, ^5^, ^6^ Therefore, ICI‐based immunotherapies have been used to treat CRC. Patients with metastatic CRC with high microsatellite instability (MSI‐H) or mismatch repair deficiency (dMMR) can benefit from ICI‐based immunotherapy because MSI‐H/dMMR tumors have high mutation rates and are highly immunogenic, which can activate anti‐tumor immune effects.^7^ In 2022, the Chinese Society of Clinical Oncology recommended immunotherapy as first‐line treatment for patients with metastatic CRC.^8^ The results of the CheckMate 142 study suggested that immunotherapy confers a survival benefit for patients with CRC.^9^ Specifically, using dual immunotherapy as the first‐line treatment for untreated patients increased the treatment‐efficacy from 60% to 69% and the complete‐remission rate from 7% to 13%. Unfortunately, most metastatic CRCs (~95%) are microsatellite stable (MSS), which is often associated with resistance to immunotherapy, probably because of the lack of immune cell infiltration into the tumor microenvironment (TME).^10^, ^11^, ^12^ Thus, MSS tumors are also known as ‘cold’ tumors. A great challenge in metastatic colorectal tumor immunotherapy is overcoming the bottleneck in MSS CRC treatment by stimulating cold MSS tumors to become ‘hot’ tumors (similar to MSH‐I/dMMR tumors).

Previous findings have shown that the cyclic GMP‐AMP synthase stimulator of interferon genes (cGAS‐STING) signaling pathway is a major mediator of DNA immune responses in vivo.^13^, ^14^ cGAS is a biosynthetic enzyme that acts as an intracellular DNA receptor, recognizes the abnormal presence of double‐stranded DNA (dsDNA) in the cytoplasm, and induces the production of type‐I interferons (IFN‐I). In turn, IFN‐1 can enhance the ability of cytotoxic T lymphocytes (CTLs) to specifically kill antigenic target cells and defend against pathogenic invasion.^15^ Notably, type‐I interferons in the TME can recruit effector T cells to kill tumor cells, thereby activating natural immunity and enhancing the anti‐tumor immune effects. Therefore, clinical studies on STING small‐molecule activators are currently being conducted. Preliminary results showed that a newly developed STING agonist (JNJ‐67544412) could bind to all major alleles of human STING(hSTING), resulting in increased levels of pro‐inflammatory factors in the nuclear plasma of tumor‐bearing mice, increased numbers of CD8^+^ T cells, and accelerated tumor cell apoptosis.^16^

Although damaged DNA is a natural agonist of the cGAS‐STING pathway, previous reports have shown that chemotherapeutic drugs can induce cellular DNA damage and sensitize patients to immunotherapy by activating the cGAS‐STING pathway and triggering an immune response. One such drug is oxaliplatin, a chemotherapeutic and non‐specific cytotoxic agent that forms cross‐links with DNA in cells, thereby inhibiting DNA replication and transcription and causing cell‐cycle arrest. Therefore, Pt‐based drugs are thought to activate natural immunity by inducing nuclear DNA (nDNA) damage.^17^ Mammalian cells also contain mitochondrial DNA (mtDNA), which is more susceptible to attack than nDNA. For example, the ZnS@BSA complex constructed by Guo et al. promoted the cellular production of reactive oxygen species (ROS), which damaged mitochondria, leading to the release of mtDNA and eventual activation of the cGAS‐STING pathway.^18^ Li et al. found that (BPA + CPI)@PLGA NPs disrupted mitochondrial metabolism in tumor cells, which caused mtDNA outflow and cGAS‐STING signaling initiation.^19^ Therefore, we speculate that simultaneous damage to both nDNA and mtDNA might better activate natural immunity.

Curcumin is a natural phenolic antioxidant with extensive anticancer effects on gastrointestinal tumors, melanoma, genitourinary tumors, breast cancer, and lung cancer.^20^, ^21^, ^22^, ^23^ Curcumin can promote Ca^2+^ release from the endoplasmic reticulum to the cytoplasm and inhibit Ca^2+^ efflux from cells, thereby causing mitochondrial Ca^2+^ overload, which can lead to mitochondrial damage‐induced cell death.^24^ However, its low solubility, poor stability, and low absorption rate have significantly hindered widespread applications. Hydroxyapatite (HAP), a substance present in the human body, has been reported to be effective in killing certain types of tumor cells via Ca^2+^ overload, including liver, osteosarcoma, glioma, lung, and breast cancer cells.^25^ Moreover, HAP can be applied as a safe and effective drug‐delivery vehicle with pH‐dependent drug release properties.^26^ Encapsulating curcumin encapsulation into nano‐HAP vehicles increased intracellular Ca^2+^ levels (due to Ca^2+^ released during HAP degradation) and enabled the tumor‐specific release of curcumin.^27^ Therefore, calcium overload‐mediated mtDNA damage was achieved.

**SCHEME 1:**
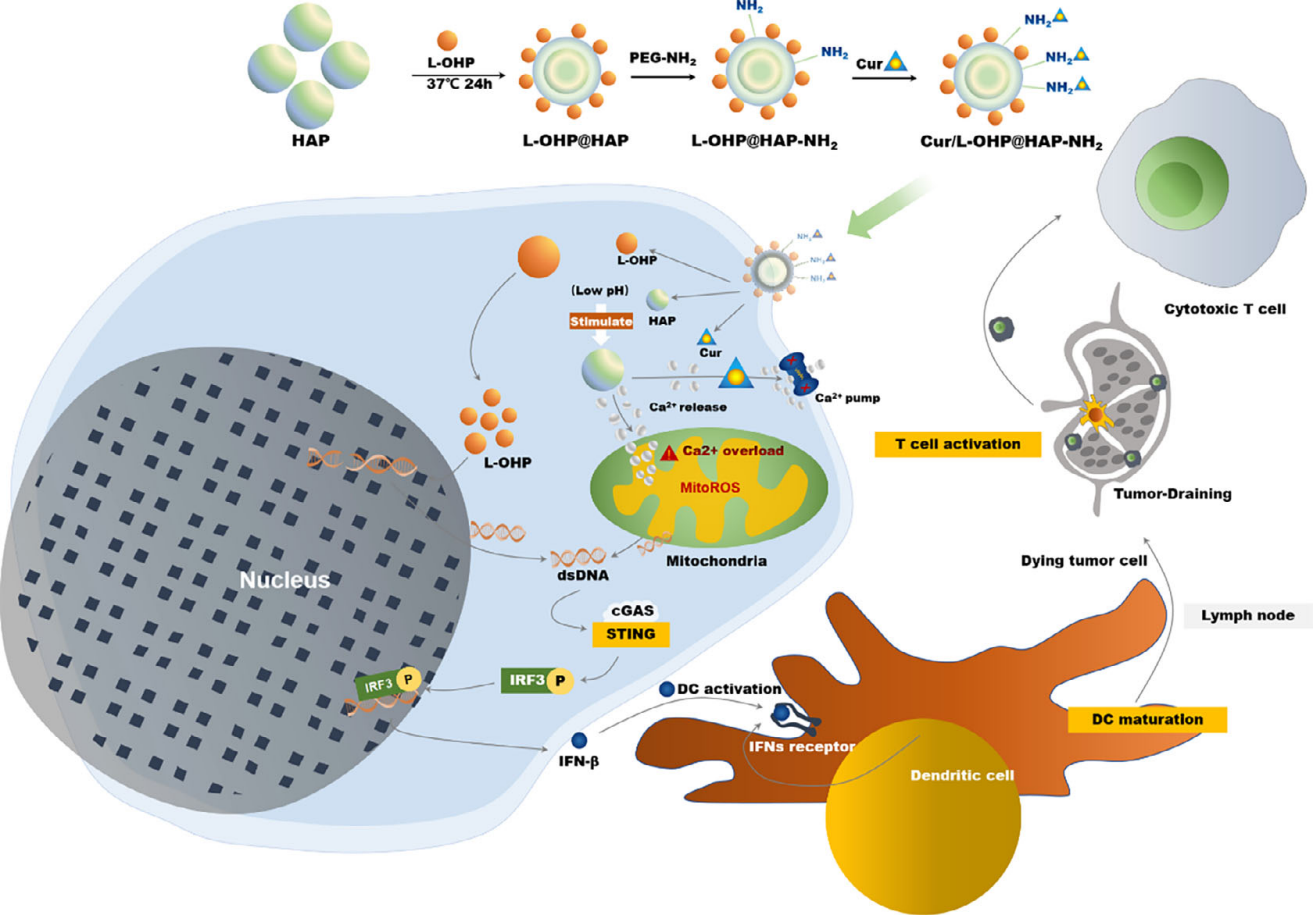
Schematic illustration of cGAS‐STING pathway activation strategy using curcumin/L‐oxaliplatin‐loaded hydroxyapatite nanaoparticles to enhance the natural immune response in colorectal cancer.

In this study, curcumin and L‐OHP‐co‐loaded HAP (Cur/L‐OHP@HAP) nanomaterials were constructed, and their anti‐tumor mechanisms and ability to activate innate immunity were explored both in vitro and in vivo. As shown in Scheme 1, HAP, a pH‐sensitive drug‐delivery vehicle, can respond to an acidic TME and release Ca^2+^, curcumin, and L‐OHP. Curcumin can further promote Ca^2+^ release from the endoplasmic reticulum to the cytoplasm and inhibit Ca^2+^ efflux, thereby synergistically enhancing Ca^2+^ overload, leading to mitochondrial damage and mtDNA release. In contrast, l‐OHP causes nDNA damage. Activation of the cGAS‐STING pathway can be efficiently triggered by the dual release of mtDNA and nDNA, stimulating interferon regulatory factor 3 (IRF3), triggering IFN‐I response, inducing dendritic cell (DC) maturation, increasing CD8^+^ T cell infiltration into tumors, and thus enhancing the immune response.

## 2 MATERIALS AND METHODS

### 2.1 Chemicals

HAP and COOH‐PEG‐COOH were purchased from MeloPEG (Shenzhen, China), and the ROS Assay Kit and Rhodamine B were purchased from Solarbio (Beijing, China). The Cell Counting Kit‐8 (CCK‐8) Kit was purchased from Dojindo (Shanghai, China), and 2‐(4‐amidinophenyl)‐6‐indolecarbamidine dihydrochloride (DAPI) and the EdU Cell Proliferation Kit were purchased from Meilun (Dalian, China). A JC‐1 Kit. The BCA Protein Assay Kit and WB Lysis Buffer were purchased from Beyotime Biotechnology (Shanghai, China). Phosphate‐buffered saline (PBS), Dulbecco's modified Eagle's medium (DMEM), fetal bovine serum (FBS), and 0.05% trypsin–EDTA were obtained from Gibco Laboratories (NY, USA).

### 2.2 Animals

Female BALB/c mice (4 weeks old) were purchased from the Animal Experiment Center of the Experimental Animal Centre of Guangdong Province and housed in an environmentally controlled breeding room with a temperature of 22 ± 2°C and a relative humidity of 70% ± 5% (Certification No. SYXK(yue)2018‐0186).

### 2.3 Synthesis of Cur/L‐OHP@HAP

To construct Cur/L‐OHP@HAP NPs, oxaliplatin (20 mg) was added to 20 mg HAP NP solution (50 mL) and continuously stirred for 24 h at 25°C. After centrifugation at 12,000×*g* for 10 min, the resulting product was washed with deionized water to obtain HAP@oxaliplatin. Then, 10 mg HAP@oxaliplatin was stirred with 10 mg PEG‐NH_2_ for 24 h to obtain oxaliplatin@HAP‐NH_2_. Next, 5 mg of curcumin was added to oxaliplatin@HAP‐NH_2_. After stirring for 24 h, Cur/L‐OHP@HAP was collected by centrifuging the mixture at 10,000×*g* for 15 min. Finally, Cur/L‐OHP@HAP was washed thrice with deionized water and stored at room temperature.

### 2.4 Characterization of Cur/L‐OHP@HAP

We observed the surface morphology and obtained elemental mapping images of Cur/L‐OHP@HAP NPs via transmission electron microscopy (TEM; JEM‐2100F, JEOL, Japan). X‐ray diffraction (XRD; Ultima IV, Rigaku, Japan), x‐ray photoelectron spectroscopy (Thermo Fisher K‐Alpha, Thermo Fisher Scientific, USA), Fourier‐transform infrared spectroscopy (FT‐IR; Nicolet IS5, Thermo Fisher Scientific), and ultraviolet–visible (UV–Vis) spectroscopy (UV‐2600, Shimadzu, Japan) were used to evaluate the Cur/L‐OHP@HAP NPs produced in this study. A Zetasizer (Zetasizer Nano‐ZS, Malvern) was used to measure the zeta potentials.

### 2.5 Cell culture

Mouse CRC cells (CT26 cells) were purchased from the American Type Culture Collection and cultured in DMEM containing 10% FBS, 100 IU/ml penicillin, and 100 mg/mL streptomycin in a 37°C incubator supplied with 5% CO_2_. When the cells reached 80%–90% confluence, they were digested and seeded the cells into plates or wells for maintenance or analysis.

### 2.6 Intracellular NP‐uptake assay

CT26 cells were incubated with Cur/L‐OHP@HAP NPs for 0–24 h and observed via flow cytometry, TEM, and confocal laser‐scanning microscopy (CLSM). For flow cytometry, the treated cells were washed with PBS, and the intracellular fluorescence intensity of Cur/L‐OHP@HAP was evaluated. Similar to our flow‐cytometric analysis, the CT26 cells were washed with PBS, fixed with 4% paraformaldehyde, stained with MitoTracker Green (30 min) and DAPI (10 min), and imaged through CLSM.

### 2.7 In vitro anti‐tumor assay

We detected cytotoxicity in CT26 cells by performing CCK‐8, 5‐ethynyl‐2′‐deoxyuridine (EdU), and Live/Dead assays. For the CCK‐8 assay, CT26 cells were seeded in a 96‐well plate and cultured for 12 h. Subsequently, various concentrations of HAP, HC (HAP@Cur), HL (HAP@L‐OHP), or HCL (Cur/L‐OHP@HAP) to each well. After 24 h, the CCK‐8 Kit was used to determine the half‐maximal inhibitory (IC_50_) values. CT26 cells were seeded in 24‐well plates and cultured for 12 h, followed by subsequent culture with HAP, HC, HL, or HCL solution for another 24 h. The treated cells were collected and stained using a Live/Dead Kit for cell‐viability analysis or an EdU Testing Kit (Keygen) for cell‐proliferation analysis.

### 2.8 Comet assay

CT26 cells were cultured in 6‐well plates for 24 h. Subsequently, HAP, HC, HL, or HCL was added, and the cells were incubated for another 24 h. Next, the treated cells were prepared as single‐cell suspensions and mixed with low‐melting point (LM) agarose at 37°C. The agarose–cell mixtures were applied to pretreated slides and exposed to lysis solution at 4°C. The lysis solution consisted of a detergent and a high‐salt solution, which removed excess cell membranes and histones. Then, the slides were immersed in gel‐electrophoresis solution, after which they were neutralized. After the LM agarose had dried completely, the slides were stained with the fluorescent dye SYBR Gold, and ~60 cells were analyzed under a fluorescent microscope to observe the extent of DNA damage.

### 2.9 Evaluation of mitochondrial function

After pre‐treating CT26 cells with HAP, HC, HL, or HCL for 24 h, the cells were collected, and mitochondrial functions were assessed using a MitoROS Kit (AAT Bioquest, Wuhan, China), a JC‐1 Kit (Keygen), and an mPTP Kit (BestBio, Shanghai, China), according to the manufacturers' instructions.

### 2.10 Ca^2+^ release of Cur/L‐OHP@HAP

CT26 cells were incubated with HAP, HC, HL, or HCL for 24 h. The cells were further incubated with the calcium‐specific dye, Fluo‐4. Ca^2+^ staining was observed using a fluorescence microscope.

### 2.11 Western blot analysis

CT26 cells were seeded into 6‐well plates and cultured for 12 h, after which HAP, HC, HL, or HCL was added to each well. After incubating the cells for 24 h, they were washed with and lysed in TAP lysis buffer for 30 min at 4°C. Proteins in the samples were resolved by sodium dodecyl sulfate‐polyacrylamide gel electrophoresis and transferred onto polyvinylidene fluoride (PVDF) membranes. The PVDF membranes were blocked with 5% bovine serum albumin (BSA) and incubated overnight at 4°C with antibodies against serca2 (1:1000, Abcam, USA), pIRF3 (1:1000, Cell Signaling Technology [CST], USA), c‐GAS (1:500, CST), IRF3 (1:1000, CST), STING (1:500, CST), and/or GAPDH (1:1000, Abcam). The membranes were washed with PBST and incubated with horseradish peroxidase‐conjugated anti‐mouse or anti‐rabbit IgG (H&L; 1:2000, Abcam) for 1 h. Finally, the membranes were developed in a dark room containing an enhanced chemiluminescence substrate.

### 2.12 Fluorescence staining

Pretreated CT26 cells were washed three times with PBS and then incubated with Fluo‐4 AM, Rhodamine 123, 3,3′‐dioctadecyloxacarbocyanine perchlorate, 2,7‐dichloride‐hydrofluorescein diacetate, calcein AM, propidium iodide (PI), MitoTracker, fluorescein isothiocyanate (FITC)‐conjugated phalloidin, or dihydroethidium for 30 min at 37°C. DAPI was used for nuclear‐localization analysis. After incubating cells with 2 μg/mL DAPI for 10 min at 37°C, they were washed three times with PBS, held in serum‐free and phenol red‐free medium, and subsequently using a fluorescence microscope (Nikon).

### 2.13 In vivo anti‐tumor effects

We constructed a mouse model using CT26 tumor cells to evaluate the effects of Cur/L‐OHP@HAP in vivo. Female BALB/c mice were injected subcutaneously with CT26 cells in the right hind limb for 10 days to initiate tumor formation. To date, the in vivo distribution of Cur/L‐OHP@HAP NPs, as CRC‐bearing mice were injected with ICG labeled Cur/L‐OHP@HAP NPs drugs through tail, and being captured using a small animal in vivo fluorescence imaging system (DIGITAL FPRCISION MEDICINE Company, Beijing, China). Furthermore, the mice were divided into five groups that were injected with saline, HAP, HC, HL, or HCL through the tail vein on days 0, 2, and 4. The tumor volumes were recorded from the first day after injection to the end of the experiment and calculated as follows: length × width × width × 0.5. The mice were sacrificed on day 15 and the tumors were measured, weighed, and fixed in 4% paraformaldehyde for immunohistochemistry (IHC) analysis, terminal deoxynucleotidyl transferase dUTP nick end labeling (TUNEL) staining, and hematoxylin and eosin (H&E) staining.

### 2.14 H&E and IHC analysis

The tissues were fixed in 4% paraformaldehyde, embedded, sectioned, and then stained with H&E for microscopic observations. For IHC staining, the sections were deparaffinized, processed for antigen retrieval, quenched to inhibit endogenous peroxidase activity, and subsequently incubated with an appropriate primary antibody. Then, the sections were incubated with anti‐mouse IgG for 30 min at 25°C, washed with Tris‐buffered saline, and incubated in 3,3‐diaminobenzidine solution for 10 min at 25°C. Finally, the sections were counterstained with hematoxylin.

### 2.15 Enzyme‐linked immunosorbent assay (ELISA) analysis

Tumor‐bearing mice were treated and sacrificed at the experimental endpoint, after which the tumor tissues were collected. The tumor tissues were suspended in an equal volume of PBS, homogenized, and centrifuged at 12,000 rpm for 10 min. The expression levels of IFN‐β, TNF‐α, and IL‐6 in tumor tissues were detected using ELISA kits for each target.

### 2.16 In vivo analysis of recruiting immune cells in tumor

After constructing the CT26 tumor model, tumor tissues and lymph nodes were obtained and digested using collagenase IV (0.3 mg/ml) for 1 h at 37°C. Single‐cell suspensions were obtained by filtration through a 70 μm mesh. The harvested cells were blocked with anti‐mouse CD16/CD32 for 15 min at 4°C, after which they were stained with eBioscience Fixable Viability Dye eFluor 506 for 15 min. The cells were then incubated with APC‐conjugated anti‐CD3, PC‐conjugated anti‐Cy7 and anti‐CD45, PerCP‐Cy5.5‐conjugated anti‐CD8, and FITC‐conjugated anti‐CD4 antibodies to assess the contents of CD4^+^ or CD8^+^ T cells in the tumors using flow cytometry, following standard protocols. After immunofluorescent staining with APC‐conjugated anti‐CD86, PE‐Cy7‐conjugated anti‐CD11c, and PE‐conjugated anti‐CD80, we examined the frequencies of mature DCs in lymph node samples via flow cytometry, using an FASCVerse instrument (Becton Dickinson, USA). The harvested tumor tissues were blocked with BSA, stained with primary antibodies (including anti‐CD3 and anti‐CD8 antibodies), and then stained with secondary fluorescent antibodies.

### 2.17 Statistical analysis

The results of our statistical analyses are presented as the mean ± SD of at least three independent experiments. Significant differences were compared using one‐way or two‐way analysis of variance. Differences were analyzed using SPSS and *p* values of <0.05 were considered to reflect statistically significant differences.

## 3 RESULTS AND DISCUSSION

### 3.1 Synthesis and characterization of Cur/L‐OHP@HAP NPs

**FIGURE 1:**
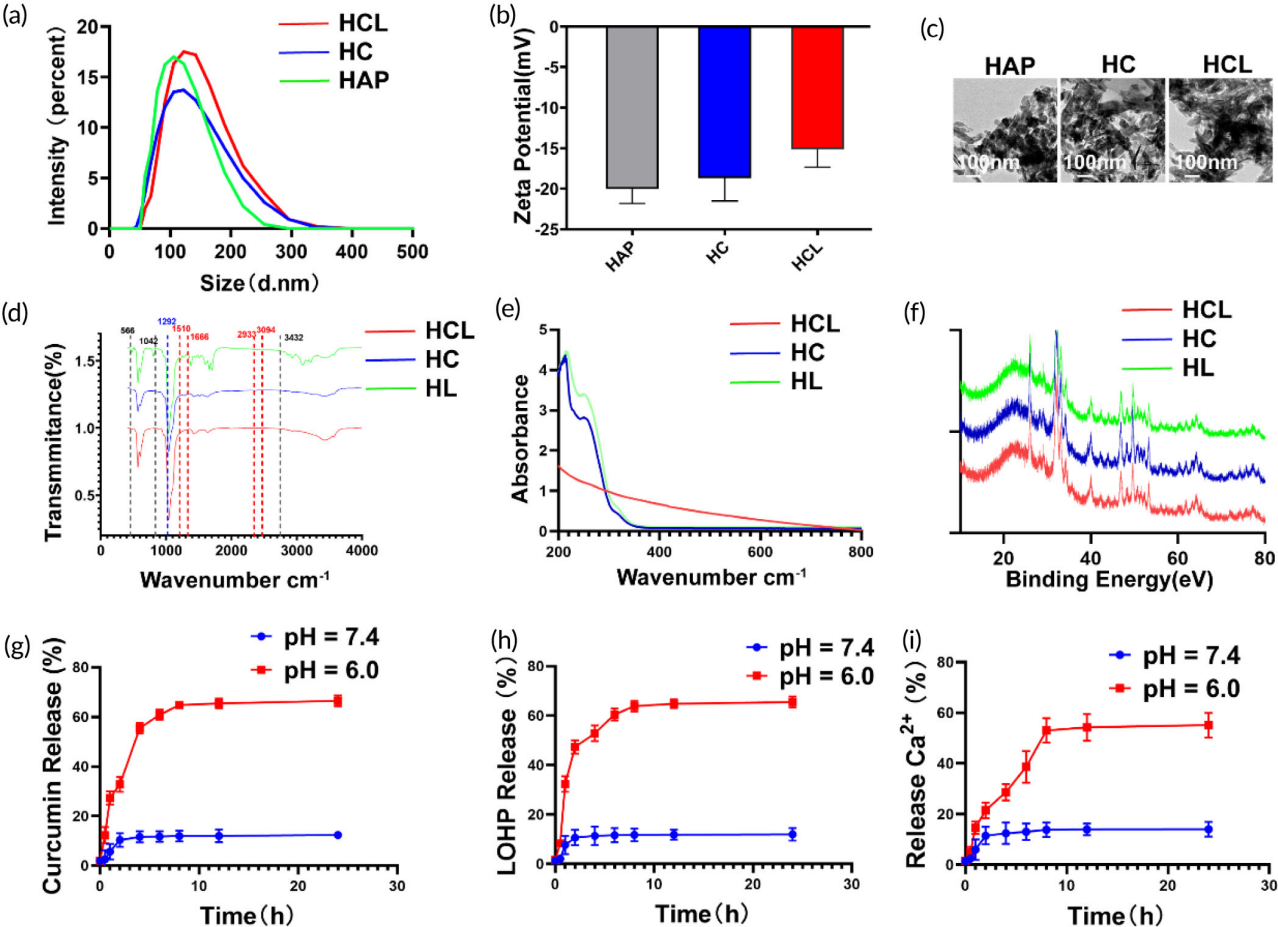
Structural characterization of HCL NPs. (a) DLS distribution of HAP, HC, and HCL NPs. (b) Zeta potentials of Synthesis process of HAP, HC, and HCL NPs. (c) TEM images of HAP, HC and HCL NPs. (d) FT‐IR spectra of HC, HL, and HCL NPs. (e) UV‐is analysis of HC, HL, and HCL NPs. (f) XRD assessment of HC, HL, and HCL NPs. (g–i) The releasing curve of curcumin, L‐OHP and Ca^2+^ of HCL NPs, at pH = 7.4 and pH = 6.0 condition.

HAP nanovehicles were constructed and loaded with curcumin and L‐OHP at a 1:2 molar ratio (defined as Cur/L‐OHP@HAP). The dynamic‐light scattering results showed that the HAP, HC, and HCL nanocomplexes were approximately 100 nm in size (Figure 1a). The zeta‐potential measurements of HAP, HC, and HCL were −20, −18, and −15 mV, respectively (Figure 1b), indicating that drug loading slightly changed the average zeta potential. Consistently, TEM images showed that HAP exhibited a rod‐like structure with a length of approximately 100 nm and that curcumin and L‐OHP loading did not appreciably influence its morphology (Figure 1c). Compared with HAP, HCL showed a new peak at 1292 cm^−1^ (which could be attributed to curcumin) and new peaks at 1510, 1666, 2933, and 3094 cm^−1^ (attributable to L‐OHP), confirming that HAP was successfully loaded with curcumin and L‐OHP (Figure 1d). Moreover, the addition of curcumin and L‐OHP inhibited new peaks with wavenumber in the UV–Vis range (Figure 1e). The encapsulation efficacy (EE) of curcumin and L‐OHP was 72% and 70%, respectively, and the loading efficacy (LE) of curcumin and L‐OHP was 41% and 26.5%. Furthermore, XRD analysis of the HCL nanoparticles showed that oxaliplatin and curcumin encapsulation did not influence the HAP crystal structure (Figure 1f). These results confirmed the successful construction of the Cur/L‐OHP@HAP nanocomposite.

A previous report showed that HAP exhibited almost complete dissolution of the deposited layer when immersed in an acidic environment,^28^ indicating that HAP can undergo biodegradation at a low pH (such as in the TME) and thereby release calcium and loaded drugs. Therefore, we studied the pH‐sensitive, drug‐release curves of curcumin, L‐OHP, and Ca^2+^ in vitro. As shown in Figure 1g–i, only 12.4% curcumin, 11.9% L‐OHP, and 15% Ca^2+^ were released within 24 h at pH 7.4 (neutral environment), whereas at pH 6.0, the amounts of curcumin, L‐OHP, and Ca^2+^ released reached up to 66.5%, 65.5%, and 60%, respectively. Tumor tissues have a weakly acidic extracellular pH (pH 6.0–6.8), whereas normal tissues have a weakly basic environment (pH 7.4).^29^ Thus, we anticipated that Cur/L‐OHP@HAP NPs could exhibit pH‐accelerated drug release in the TME.

### 3.2 In vitro cellular uptake and anti‐tumor effects of Cur/L‐OHP@HAP NPs

**FIGURE 2:**
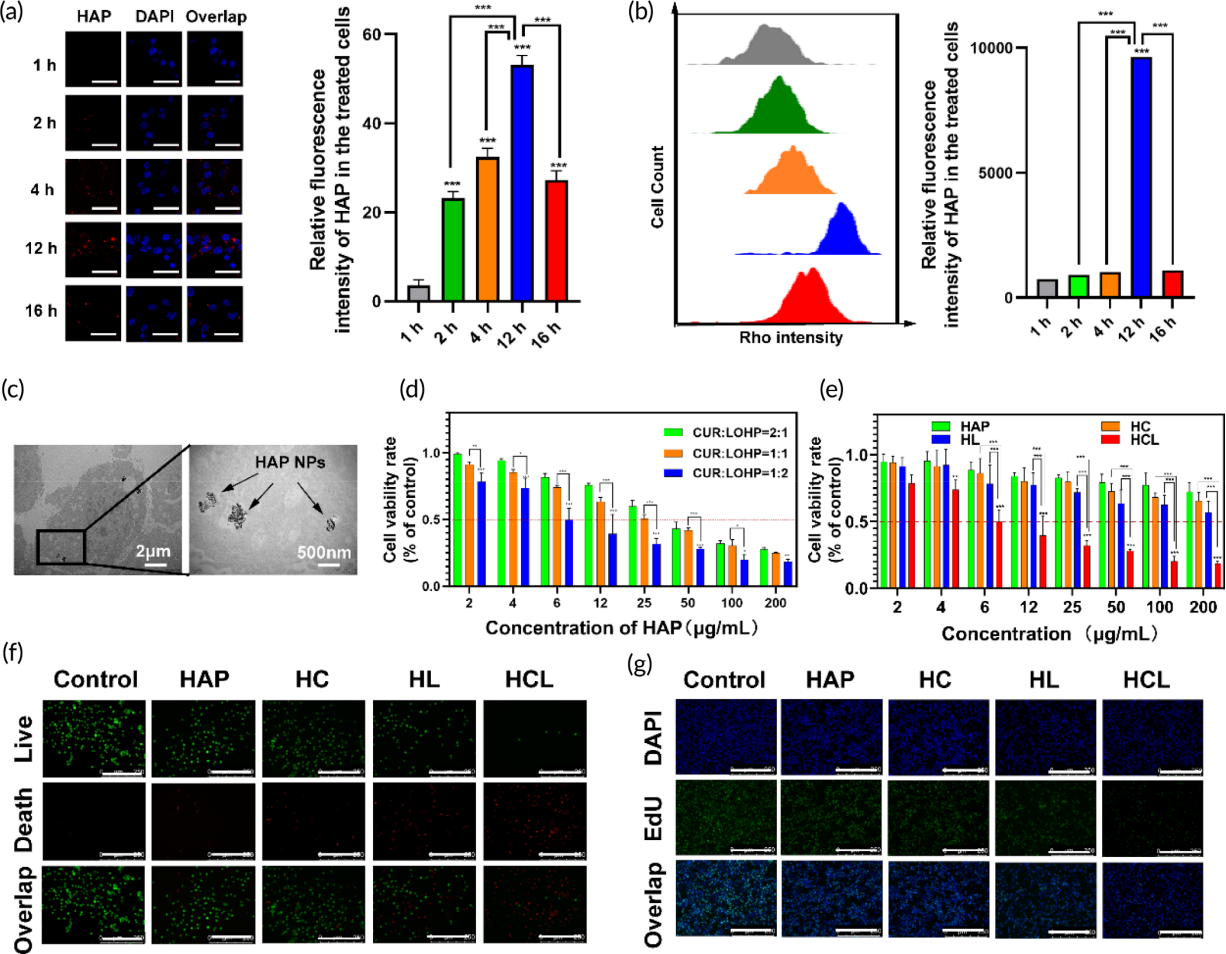
Cellular uptake and in‐vitro antitumor effect of HCL NPs. (a) CLSM images (left) and fluorescence statistics of CT26 cells treated with RhB‐labeled HCL NPs, scale bar: 50 μm. (b) Flow cytometry images and fluorescence analysis of the intracellular RhB fluorescence intensity inside CT26 cells treated with RhB‐labeled HCL NPs. (c) TEM images of CT26 cells treated with HCL NPs for 4 h, scale bar: 2 μm/500 nm. (d) Cell viabilities of CT26 cells treated with various concentrations of HCL solutions, at different ratios of curcumin and LOHP. (e) Cell viabilities of CT26 cells treated with different HAP, HC, HL, and HCL concentrations. (f) Representative live/dead stained images of CT26 cells after incubation with HAP, HC, HL, and HCL NPs (cells in green are alive and cells in red are dead), scale bar: 50 μm. (g) Representative EdU stained images of CT26 cells after incubation with HAP, HC, HL, and HCL NPs, scale bar: 50 μm. *p < 0.05, **p < 0.01, ***p < 0.001.

Nanotechnology‐based drug‐delivery systems have emerged as promising alternatives to conventional chemotherapy strategies due to their efficient drug‐delivery properties.^30^ We expected that HAP would enable efficient intracellular delivery of curcumin and L‐OHP to CRC cells. Rhodamine B (red) was used to label the Cur/L‐OHP@HAP NPs, and CLSM and flow cytometry were used to detect Cur/L‐OHP @HAP NPs uptake in CT26 cells by monitoring the fluorescence intensity of rhodamine B. As shown in Figure 2a, almost no red fluorescence was observed in CT26 cells after 1 h, after which a significant increase in the intracellular rhodamine B fluorescence was observed, with the highest cellular‐uptake efficiency found at 12 h, in agreement with the flow cytometry results (Figure 2b). Moreover, after 4 h of Cur/L‐OHP @HAP treatment, TEM results of CT26 cells showed black particles in the cytoplasm (Figure 2c). Therefore, we speculated that the cellular‐uptake efficiency of Cur/L‐OHP@HAP was highest at 12 h. We also have proved the in vivo distribution of Cur/L‐OHP@HAP NPs, as CRC‐bearing mice were injected with ICG labeled Cur/L‐OHP@HAP NPs. As shown in Figure S1, a significant fluorescence signal accumulated in the tumor in the Cur/L‐OHP@HAP‐treated mice and peaked at 8 h, while no significant fluorescence was observed in the tumor site of CRC‐bearing mice, which confirming that the passive tumor‐targeting abilities through enhanced permeability and retention (ERP) effect.

To assess the anti‐tumor effect, CCK‐8 assays were performed to detect the viability of CT26 cells after incubation with HAP, HC, HL, and HCL solutions at different concentrations. As shown in Figure 2d, the survival rates of CT26 cells in the HC, HL, and HCL groups decreased significantly with increasing concentrations, when compared with those in the HAP group. HCL showed the most powerful anticancer effect on the CT26 cells, indicating that the combination of curcumin and L‐OHP (1:2) increased the killing efficiency. The anti‐proliferative capacity of HCL prepared with different curcumin: L‐OHP ratios (1:2, 1:1, and 2:1) has been studied previously. Our CCK‐8 assay results (Figure 2e) showed that the strongest inhibitory effect on CT26 cells occurred when the curcumin: OHP ratio was 1:2 with an IC_50_ value of ~6 μg/ml, when compared to the inhibitory effects observed at the other two ratios (1:1 and 2:1). The above data suggest that the addition of HAP, Cur, and L‐OHP exhibited cancer‐cell cytotoxicity, where a higher dose of L‐OHP exerted a better anti‐tumor effect.

To further understand the anti‐tumor effects of our NPs, calcein‐AM (green fluorescence in living cells) and PI (red fluorescence in dead cells) were used to stain CT26 cells after different treatments. The Live/Dead assay results (Figures 2f and S2) showed that the percentages of dead cells in the HCL‐, HC‐, and HL‐treated groups were 26%, 19%, and 5%, respectively, which were at least 3‐fold higher than that of control group, demonstrating that HCL treatment was more effective at killing CT26 cells than the other treatments. EdU, a thymidine analog that can intercalate into replicating DNA molecules instead of thymidine, was used to detect cell proliferation. The results (Figures 2g and S3) showed that the HCL‐treated group had the lowest number of EdU‐positive cells (28.2%) compared with the control (60%), HAP (58.2%), HC (50%), and HL (42%) groups. In conclusion, HCL potently killed CT26 cells in vitro; however, the underlying anti‐tumor mechanism of HCL remains unclear.

### 3.3 Cur/L‐OHP@HAP NPs induced nDNA and mtDNA damage

**FIGURE 3:**
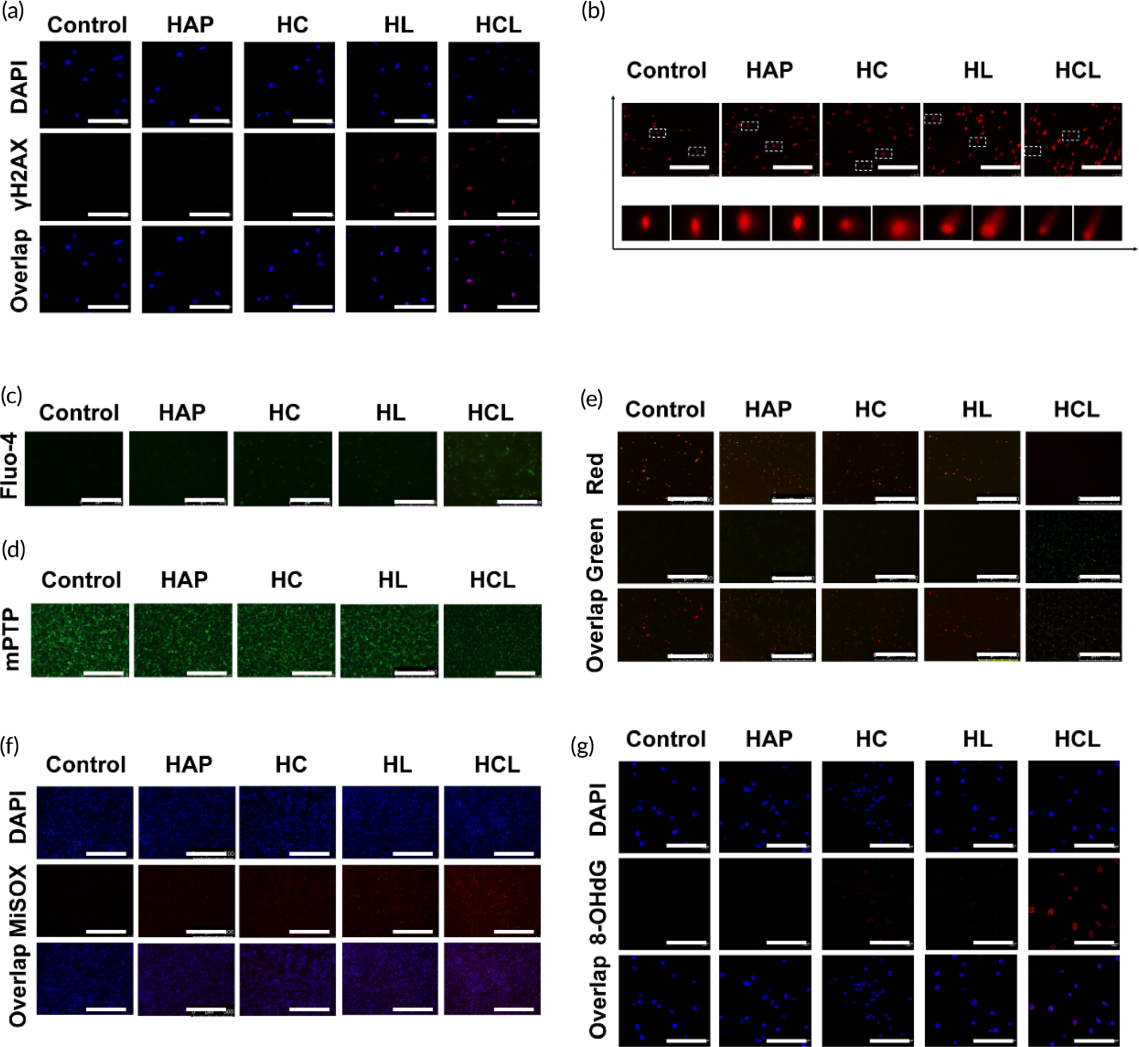
Dual nDNA and mitoDNA damage properties of HCL NPs. (a‐b) γH2AX and comet immunostaining used to evaluate the nDNA oxidation levels, scale bar: 50 μm. (c) CLSM images of Ca^2+^ (refer to Fluo‐4) in CT26 cells. Scale bar: 50 μm. (d) mPTP images of CT26 cells, detected by fluorescence inverted microscopy. Scale bar: 50 μm. (e) Fluorescence image of CT26 cells with JC‐1 after various treatments. Scale bar: 50 μm. (f) CLSM images of mitoROS (refer to MiSOX) of CT26 cells. Scale bar: 50 μm. (g) Representative immunofluorescence images showing mitoDNA oxidation levels inside treated CT26 cells stained with 8‐OHdG (red), and DAPI (blue), scale bar: 50 μm. *p < 0.05, **p < 0.01, ***p < 0.001.

Nucleolar DNA has been shown to be a key target for oxaliplatin‐induced cytotoxicity. γ‐H2AX is closely associated with cellular DNA double strand breaks (DSBs) and is, thus, used as a marker of DNA DSBs. High γ‐H2AX expression during DNA repair reflects DNA damage to some extent.^31^ To assess whether L‐OHP damages nDNA, γ‐H2AX protein expression was measured. As shown in the CLSM images (Figures 3a and S4), γ‐H2AX expression was significantly higher in the HL and HCL groups. Additionally, comet assays were performed to detect nDNA strand breaks. When cellular DNA is damaged, it leaves the nucleus during electrophoresis and migrates to form a “comet”‐like image, whereas the undamaged DNA portion remains spherical.^32^ As shown in Figures 3b and S5, the edge of the comet tail was smooth and the nucleus was intact in control group, whereas both the HL‐ and HCL‐treated groups showed clear comet‐like images, and the frequency of DNA strand breakage was up to 80%. These results verified that L‐OHP within the HL and HCL NPs induced significant DNA damage in CT26 cells.

Xia et al. discovered that intracellular HAP can undergo biodegradation in an acidic environment and thereby release calcium.^28^ To explore this, we used a Fluo‐4 AM probe to detect the production of free Ca^2+^ in the cytoplasm of CT26 cells. The results revealed almost no green fluorescence in the control group, whereas weak green fluorescence was observed in the HAP‐treated group, which may have reflected calcium‐ion release from HAP into CT26 cells (Figures 3c and S6). In addition, curcumin can inhibit Ca^2+^ efflux from cells, causing Ca^2+^ overloaded.^24^ Our CLSM images showed that the HC and HCL groups had stronger green fluorescence (higher intracellular free Ca^2+^ concentrations), with an approximately 8‐fold‐increase over that in the control group. We further examined sarcoplasmic reticulum Ca^2+^‐ATPase (serca2) expression in CT26 cells after treatment with HAP NPs. Western blotting results (Figure S7) showed that serca2 expression was significantly lower in the HC and HCL groups, which verified previous findings showing that curcumin promoted endoplasmic reticulum Ca^2+^ release and inhibited sarcoplasmic reticulum Ca^2+^‐ATPase and cytosolic Ca^2+^ efflux, which increased the Ca^2+^ concentration in CT26 cells. Taken together, these findings indicate that HAP induced Ca^2+^‐dependent cell death and that curcumin amplified the Ca^2+^‐dependent overload.

Interestingly, mitochondria have been reported to be important direct targets of Ca^2+^; therefore, we considered whether HAP‐released Ca^2+^ might help maintain mitochondrial homeostasis. Previous results have shown that mitochondrial dysfunction is closely related to cell viability.^33^ The mitochondrial permeability‐transition pore (mPTP) is composed of a group of protein complexes that exist between the inner and outer membranes of mitochondria and help maintain the mitochondrial‐membrane potential (MMP) and ion balance between the inside and outside of cells.^34^ As illustrated in Figures 3d and S8, the green‐fluorescence intensity, representing mitochondrial calcein‐AM aggregation via mPTP, decreased in the presence of HAP‐dependent NPs, indicating that increased mPTP opening occurred in mitochondria with higher Ca^2+^ levels. Furthermore, the mPTP fluorescence intensities were significantly lower in the HC and HCL groups than in the free HAP group. When mPTP opening occurs, HAP may result in MMP depolarization and more severe mitochondrial damage. To further evaluate the role of HCL in the mitochondria of CT26 cells, a JC‐1 fluorescent probe was used as a cationic anabolic dye, such that a decrease in the mitochondrial MMP could be indicated by decreased red fluorescence or a decrease in the red: green ratio. As shown in Figures 3e and S9, the red: green fluorescence ratios in the HAP‐treated and control groups were not significantly different. In contrast, the red fluorescence was more attenuated in the HC‐ and HL‐treated groups. The red fluorescence was significantly attenuated and the green fluorescence increased most significantly in the HCL‐treated group, with the red: green fluorescence ratio of the HCL‐treated group being 1/12 of that of the control group. One possible explanation for these findings is that mitochondria play a regulatory role by sequestering excess Ca^2+^ from the cytoplasm, and calcium overload in the mitochondria leads to a decrease in the MMP, which indicates that Cur/L‐OHP@HAP NPs can cause mitochondrial damage in CT26 cells.

In addition, MitoSOX is a hydroethidine compound that targets mitochondria, relies on Mitochondrial permeability transition pores (mPTP) accumulation in mitochondria, and emits red fluorescence upon oxidation and binding to mitochondrial DNA^35^; thus, it is used to detect the production of mitochondrial ROS (referred to as mitoROS). As shown in Figures 3f and S10, red fluorescence was not observed in the control group, faint red fluorescence was observed in the HC and HL groups, and relatively strong red fluorescence was observed in the HCL‐treated group. The intensity of red fluorescence in the HCL‐treated group was approximately 4‐fold higher than that in the HC‐treated‐ and HL‐treated groups, demonstrating that a relatively large amount of intra‐mitoROS was produced in HCL‐treated cells. Owing to a lack of histone protection and poor self‐repair ability, mtDNA is easily attacked by mitoROS and is converted into oxidized mitochondrial DNA (Ox‐mtDNA).^36^ We used 8‐OHdG (red) to label Ox‐mtDNA in CT26 cells and assessed the effects of HCL on Ox‐mtDNA damage. As shown in Figures 3g and S11, strong red fluorescence of Ox‐mtDNA was most evident in the HCL‐treated group, with the red fluorescence intensity being approximately 8‐fold, 4‐fold, and 4‐fold higher than that of the HAP‐treated, HC‐treated, and HL‐treated groups, respectively, demonstrating that mtDNA damage was most severe in HCL‐treated CT26 cells.

### 3.4 In vivo anti‐tumor efficiency of Cur/L‐OHP@HAP NPs

**FIGURE 4:**
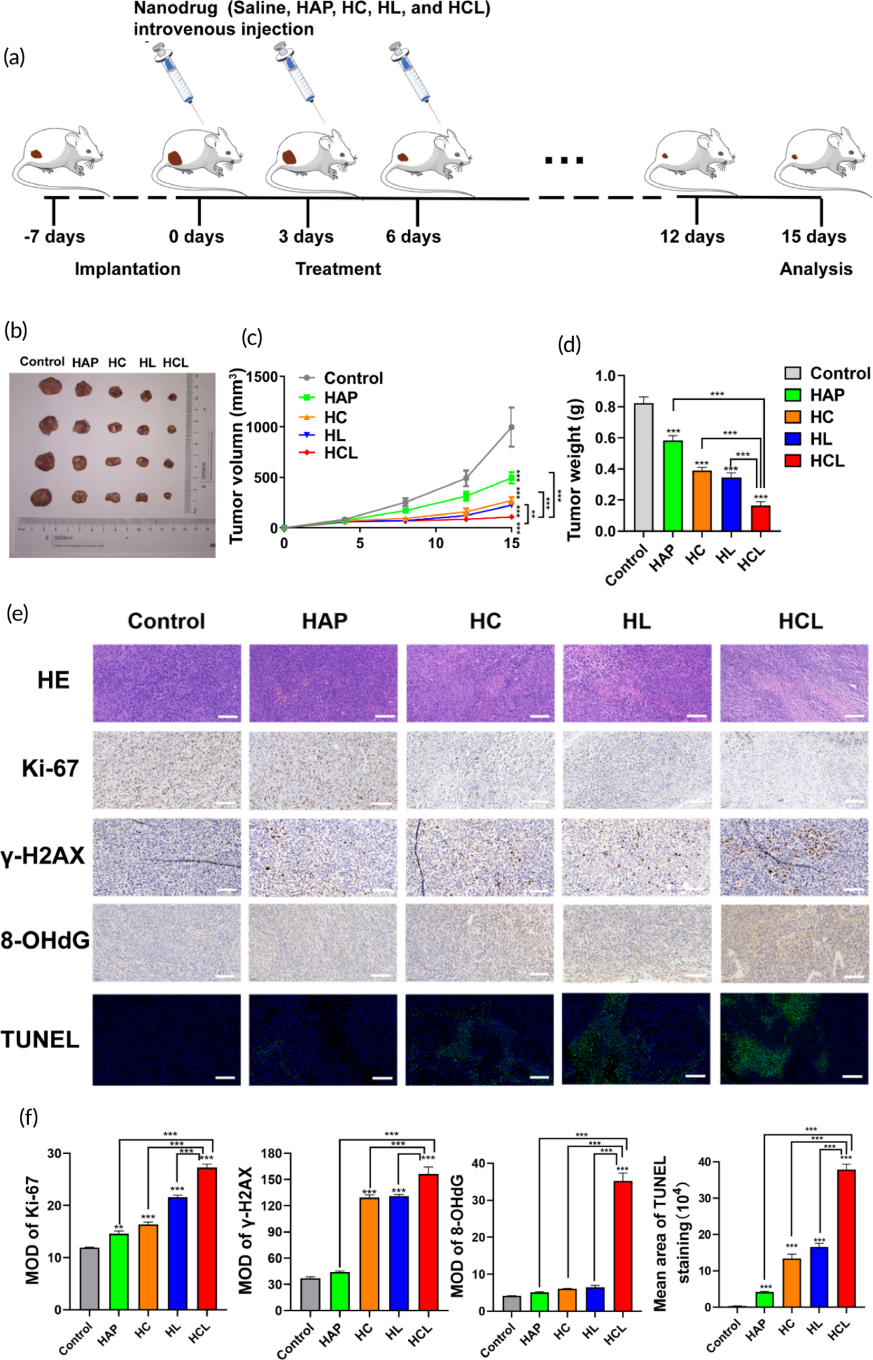
In‐vivo anti‐tumor effect of HCL NPs. (a) Illustration of the treatment schedule used to evaluate the effects of our‐designed HAP‐based NPs. (b) Photograph of the tumors on day 15 when the mice were euthanized. (c, d) Tumor‐growth curves and tumor weight on CT26‐bearing mice. (e) H&E, Ki‐67, γ‐H2AX, 8‐OHdG, and TUNEL staining of the tumors extracted from mice after various treatments. (f) Quantitative charts of Ki67, γ‐H2AX, 8‐OHdG, and TUNEL staining, scale bar: 100 μm. *p < 0.05, **p < 0.01, ***p < 0.001.

Given that we demonstrated that Cur/L‐OHP@HAP NPs can effectively inhibit tumor cell growth and damage both nDNA and mtDNA in tumor cells in vitro, we further examined its anticancer effects in vivo. Tumor‐bearing mice were randomly divided into the following five groups: (1) control, injected with saline; (2) HAP; (3) HC; (4) HL; and (5) HCL. Tumor volumes and weights were recorded from the first day after injection until the end of the experiment. The mice were euthanized on the 15th day and the tumors were excised, measured, and weighed (Figure 4a). The results (Figure 4b–d) showed that the tumor volumes in the HCL group were 87% less than those in the saline group after 15 days of treatment. The tumor volumes in the HAP, HC, and HL groups were 74%, 49%, and 45% lower than those in the saline group, respectively. By measuring the tumor weights of mice in each group after 15 days, we found that the tumor weights of the HCL group were significantly lower than those of the other groups. These results indicated that tumor growth in HCL‐treated mice was significantly inhibited, demonstrating that HCL NPs effectively inhibited tumor growth.

To further confirm the potential in vivo therapeutic effect of Cur/L‐OHP@HAP NPs, we investigated the proliferation, apoptosis, and lesion planes of tumor tissues after different treatments using paraffin‐embedded tumor sections and examined tumor cell apoptosis and proliferation at the end of treatment by performing H&E, Ki‐67, and TUNEL staining (Figure 4e, f). Ki‐67 is an important cell proliferation‐related protein,^37^ the expression of which correlated positively with tumor growth and showed the lowest expression in the HCL group, indicating that HCL can inhibit tumor cell proliferation. TUNEL assays showed the highest degree of apoptosis in the HCL‐treated group, indicating that the HCL NPs induced apoptosis and had anti‐tumor effects.

Next, we detected γ‐H2AX and 8‐OHdG expression to further explore the extent of mtDNA and nDNA damage in vivo. Consistent with the results of the in vitro experiments, both γ‐H2AX and 8‐OHdG expression were significantly higher in the HCl‐treated group, which reinforces our conclusion that Cur/L‐OHP@HAP NPs induced nDNA and mtDNA damage in vivo.

We have also conducted the expression of cGAS‐STING pathway‐related proteins in tumor tissues, after being treated with different drugs. Figure S12 showed that cGAS and STING protein‐expression levels were significantly upregulated after HC, HL, and HCL treatment, particularly in the HCl‐treated group, suggesting that HCL successfully activated STING. IRF3 phosphorylation was most significantly different in the HCL group, when compared with the other groups, and was accompanied by IFN‐β upregulation, demonstrating that the HCL group noticeably activated the cGAS‐STING signaling pathway. These results consistently demonstrate the activation of cGAS‐STING signaling pathway in vivo.

### 3.5 Cur/L‐OHP@HAP NPs triggered nDNA and mtDNA damage and induced cGAS/STING‐mediated innate immunity against CRC

**FIGURE 5:**
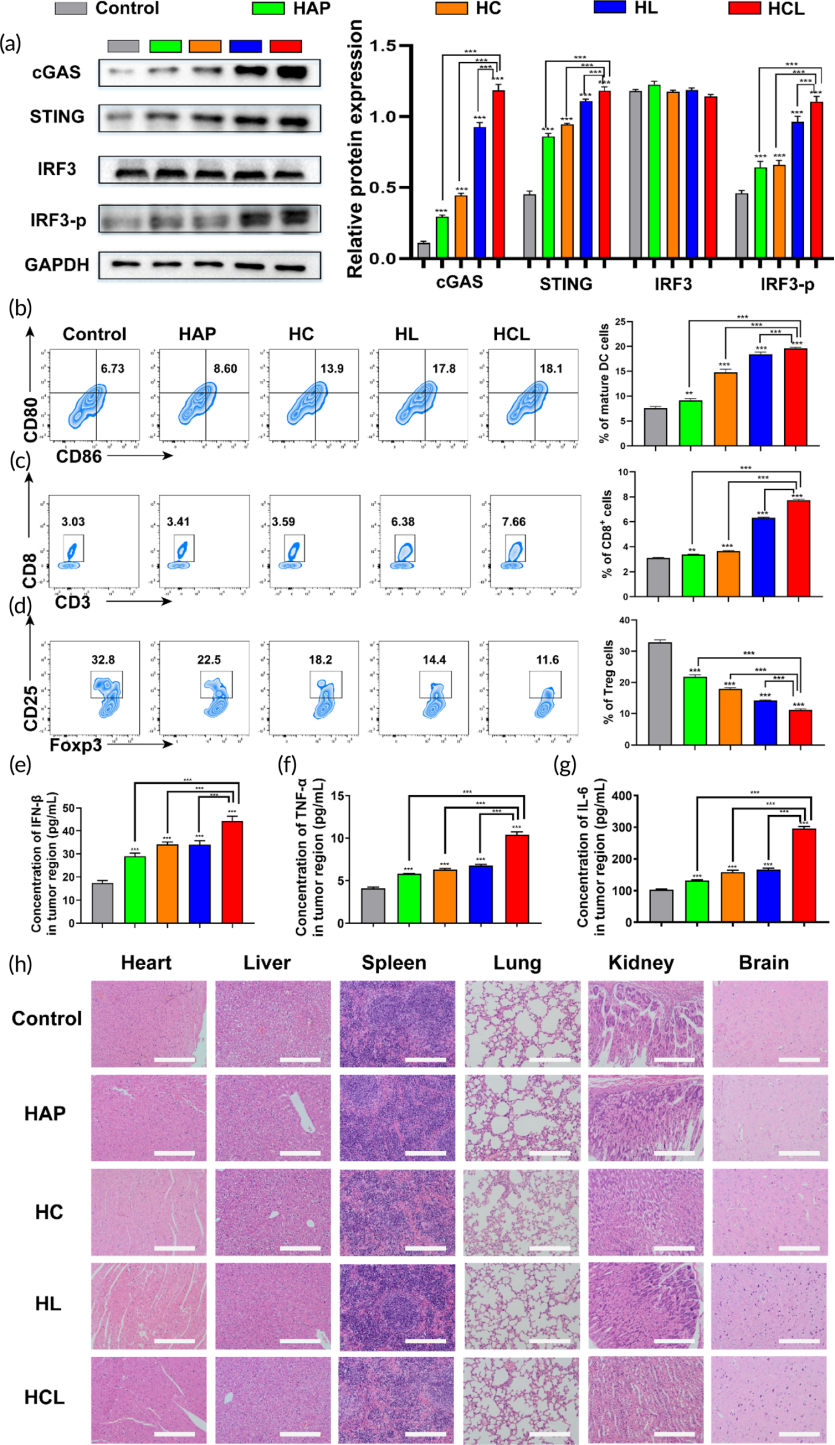
Innate immunotherapeutic effect of HCL NPs. (a) Western blot analysis for proteins related to the cGAS/STING signaling pathway, after CT26 cells being treated with HAP, HC, HL and HCL NPs for 24 h. (b–d) Flow cytometry analysis of DCs cells (CD45^+^CD11c^+^CD80^+^CD86^+^) in tumor‐draining lymph nodes, CD8^+^ T cells (CD45^+^CD3^+^CD8^+^) and Treg cells (CD45^+^CD3^+^CD4^+^CD25^+^FOXP3^+^) in tumor samples extracted from mice given treatments. (e–g) IFN‐β, TNF‐α, and IL‐6 levels in tumor samples. (h) HE staining of major organs extracted from mice after various treatments, scale bar: 100 μm. *p < 0.05, **p < 0.01, ***p < 0.001.

Tumor immunotherapy has rapidly progressed and revolutionized oncology‐treatment paradigms.^38^ Currently, low response rates remain the biggest obstacle in tumor immunotherapy.^39^ The results of recent studies have identified the cGAS/STING pathway as important for recognizing damage‐associated molecules and abnormal intracellular DNA synthesized when pathogen‐derived DNA and aberrant cytosolic dsDNA are recognized by cGAS.^40^ Subsequently, cGAMP activates the transcription factors IRF3 and nuclear factor‐kappa B through binding to the STING protein, stimulates type‐I IFN expression, and finally triggers innate immunity.^41^ As demonstrated above, HCL treatment can cause dual nDNA and mtDNA damage. We performed western blot analysis by extracting proteins from the treated groups to determine whether mtDNA and nDNA released into the cytoplasm activated the cGAS/STING signaling pathway. The western blot results (Figure 5a,e) showed that cGAS and STING protein‐expression levels were significantly upregulated after HC, HL, and HCL treatment, particularly in the HCL‐treated group, suggesting that HCL successfully activated STING. IRF3 phosphorylation was most significantly different in the HCL group, when compared with the other groups, and was accompanied by IFN‐β upregulation, demonstrating that the HCL group noticeably activated the cGAS‐STING signaling pathway. These results demonstrate that the effect of dual DNA damage is superior to nuclear or mitochondrial damage alone. Previous data also demonstrated that activation of the cGAS‐STING pathway promoted the expression and release of inflammation‐related factors, which help DCs present antigens to T and B cells and trigger innate immunity, accompanied with higher expression of cytokines such as IL‐6 and TNF‐a.

DCs are important for the production of type‐I interferons and are key antigen‐presenting cells for the maintenance of intrinsic immunity and the activation of acquired immunity.^42^ To determine whether HCL treatment could promote DC maturation and induce a stronger immune response, we used flow cytometry to detect markers of DC maturation and the specific expression of CD80 and CD86 on the cell surface. As shown in Figure 5b–d, HCL treatment induced remarkably higher CD80 and CD86 on DCs than control treatment, demonstrating that DC maturation was enhanced in the HCL‐treated group. Importantly, CD8^+^ T cells are crucial T lymphocytes for the immune response to anti‐tumor therapy. CD8^+^ CTLs kill cells by secreting perforin and granzyme B and play key roles in maintaining CD8^+^ CTL responses. CD4^+^CD25^+^FOXP3^+^ regulatory T (Treg) cells have unique immunomodulatory effects on tumor effector T cells have immunosuppressive effects, mediating tumor immune tolerance and escape. A significant increase in Treg cells has been found in various malignancies and is associated with a poor prognosis.^43^ Our flow‐cytometric analysis showed that significantly more CD8^+^ and CD4^+^ cells were present in tumors in the HCL group than in the control group and the other groups and that the frequency of CD4^+^CD25^+^FOXP3^+^ Treg cells in the HCL‐treated group was significantly lower. These results demonstrated that HCL treatment further enhanced the infiltration of immune cells and relieved the immunosuppressive function of Tregs. Activation of the cGAS‐STING pathway also stimulated a pro‐inflammatory response, leading to a high release of inflammatory cytokines, and our ELISA results (Figure 5f,g) revealed high secretion of relevant pro‐inflammatory cytokines, including IL‐6 and TNF‐α. These results collectively demonstrate that innate and tumor‐specific immune responses were activated. To support the clinical translation of our designed NPs, we treated BALB/c mice with saline, HAP, HC, HL, or HCL. No obvious damage was observed in H&E sections obtained from major organs, including the heart, liver, spleen, lungs, and kidneys (Figure 5h). These results showed that HCL NPs demonstrated good biosafety and are promising drug‐delivery composite materials.

## 4 CONCLUSIONS

In summary, we constructed a nanodrug delivery system, Cur/L‐OHP@HAP, that involves Cur and L‐OHP co‐loaded into HAP NPs. When the NPs entered the TME, HAP released curcumin and L‐OHP due to stimulation by the acidic TME. Curcumin can promote mitochondrial Ca^2+^ overload, leading to mitochondrial damage and mtDNA release, which can cause nuclear damage and nDNA release. Our experiments showed that mtDNA and nDNA release induced by the nanocomposites can greatly stimulate the cGAS‐STING pathway, induce stronger innate immunity, and achieve more significant tumor‐inhibition rates (up to 87%) than HC or HL treatment alone. In this study, we achieved effective inhibition of tumor growth through enhanced immunotherapy, which provides a potential clinical application for promoting anti‐tumor immunotherapy (Scheme 1).

**Abbreviations**
BSA: bovine serum albumin
CCK‐8: cell counting kit‐8
cGAS‐STING: cyclic GMP‐AMP synthase‐stimulator of interferon genes
CRC: colorectal cancer
CST: cell signaling technology
CTL: cytotoxic T lymphocyte
CTLA4: cytotoxic T‐lymphocyte associated protein 4
Cur: curcumin
Cur/L‐OHP@HAP NP: hydroxyapatite nanoparticles co‐loaded with curcumin and L‐oxaliplatin
DAPI: 2‐(4‐amidinophenyl)‐6‐indolecarbamidine dihydrochloride
DC: dendritic cell
DMEM: Dulbecco's modified Eagle's medium
dMMR: mismatch repair deficiency
dsDNA: double‐stranded DNA
EdU: 5‐ethynyl‐2′‐deoxyuridine
ELISA: enzyme‐linked immunosorbent assay
FBS: fetal bovine serum
FITC: fluorescein isothiocyanate
FT‐IR: Fourier‐transform infrared spectroscopy
H&E: hematoxylin and eosin
IC50: half‐maximal inhibitory
ICIs: immune‐checkpoint inhibitors
IFN: interferon
IHC: immunohistochemistry
IL: interleukin
IRF3: interferon regulatory factor 3
LM: low‐melting point
mitoROS: mitochondrial ROS
DSBs: DNA double strand breaks
MMP: mitochondrial‐membrane potential
mPTP: mitochondrial permeability‐transition pore
MSI‐H: high microsatellite instability
MSS: microsatellite stable
mtDNA: mitochondrial DNA
nDNA: nuclear DNA
Ox‐mtDNA: oxidized mitochondrial DNA
PBS: phosphate‐buffered saline
PD‐1: programmed cell death 1
PVDF: polyvinylidene fluoride
ROS: reactive‐oxygen species
TEM: transmission electron microscopy
TME: tumor microenvironment
TNF: tumor necrosis factor
Treg: regulatory T
TUNEL: terminal deoxynucleotidyl transferase dUTP nick end labeling
UV–Vis: ultraviolet–visible
XRD: x‐ray diffraction

## AUTHOR CONTRIBUTIONS

**Yao Xiao:** Data curation (lead); investigation (lead); writing – original draft (lead). **Guohu Guo:** Data curation (equal); resources (equal). **Huaiming Wang:** Investigation (equal); methodology (equal). **Bin Peng:** Data curation (equal); validation (equal). **Yinglin Lin:** Investigation (equal); validation (equal). **Gaowen Qu:** Validation (equal). **Ben Li:** Investigation (equal); validation (equal). **Zhaojun Jiang:** Validation (equal). **Fan Zhang:** Methodology (equal); resources (equal); validation (equal). **Jiaming Wu:** Funding acquisition (equal); methodology (equal); resources (equal); supervision (equal). **Min Liang:** Conceptualization (lead); funding acquisition (lead).

## FUNDING INFORMATION

This work was supported by grants from Key Laboratory of Guangdong Higher Education Institutes (2021KSYS009), Guangzhou Science and Technology Plan Project (202102010128, 202102010100, 2023A03J0820), National Natural Science Foundation Youth Fund Project (82203359), Guangdong Provincial Basic and Applied Basic Research Fund Regional Joint Fund Youth Project (2021A1515110976), Undergraduate Innovation Ability Improvement Program of Guangzhou Medical University (2022JXA005, 2022JXA006), Jiaxing Science and Technology plan Project‐Research on technological innovation of people's livelihood (2023AY31005), 2023 Jiaxing city and provinces to build medical key disciplines‐Oncology (2023‐SSGJ‐001).

## CONFLICT OF INTEREST STATEMENT

The authors declare no competing financial interest.

### PEER REVIEW

The peer review history for this article is available at https://www.webofscience.com/api/gateway/wos/peer‐review/10.1002/btm2.10610.

## ETHICS STATEMENT

All the animal experiments were conducted in accordance with the guidelines and the ethical standards of the Institutional Animal Care and Use Committee of SYXK(yue)2018‐0186.

## Supporting information


**DATA S1.** Supporting Information.

## Data Availability

The datasets used during the present study are available from the corresponding author upon reasonable request.
