# Supplementary material for: Curcumin/L‐OHP co‐loaded HAP for cGAS‐STING pathway activation to enhance the natural immune response in colorectal cancer
Source: Bioeng Transl Med. 2023 Oct 14;9(1):e10610. doi: 10.1002/btm2.10610 (PMC10771561; doi:10.1002/btm2.10610)
Supplement: Supplementary file 1 — DATA S1. Supporting Information. [file BTM2-9-e10610-s001.docx]

**Supporting Information**


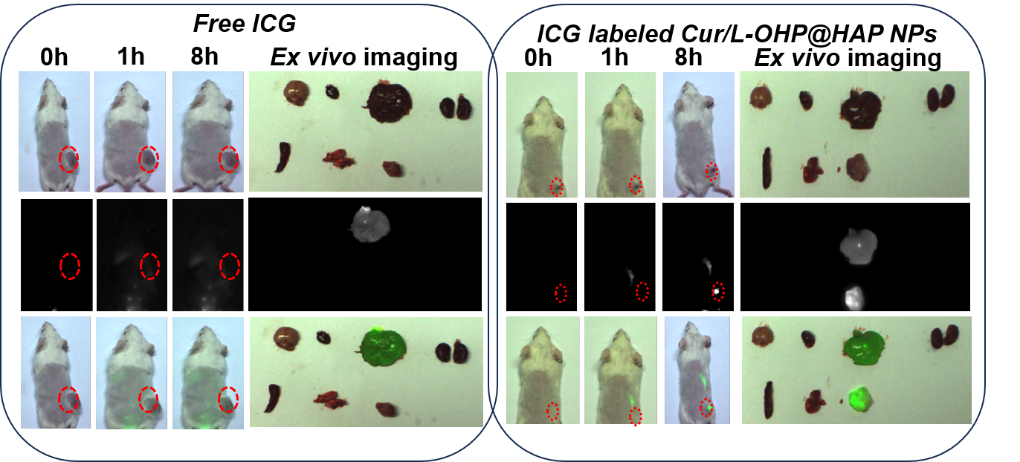


**Figure S1.** The *in vivo* distribution of ICG and ICG labeled Cur/L-OHP@HAP NPs in CRC bearing mice.


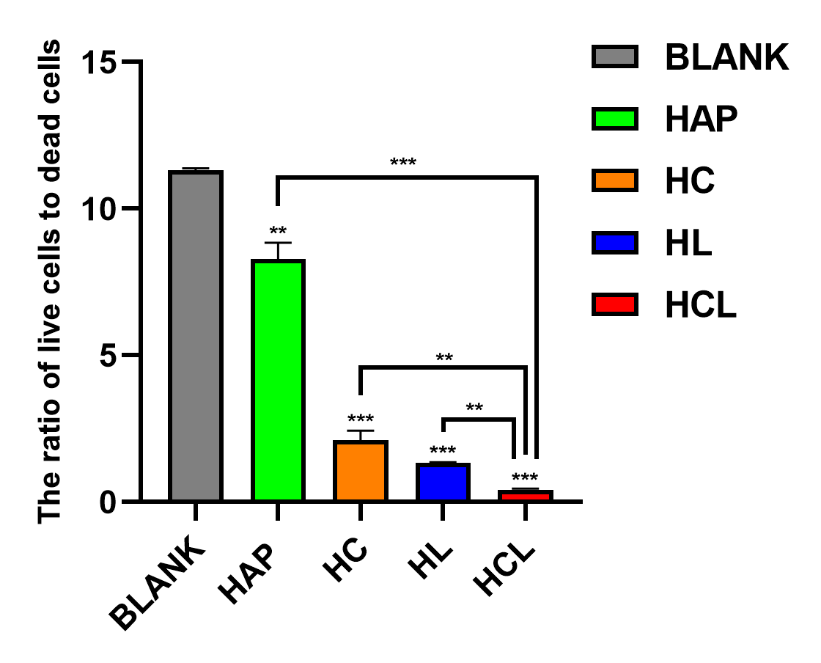


**Figure** **S2.** The statistics live/death results of CT26 cells after incubation with HAP, HC, HL and HCL NPs, **P* < 0.05, ***P* < 0.01, ****P* < 0.001.


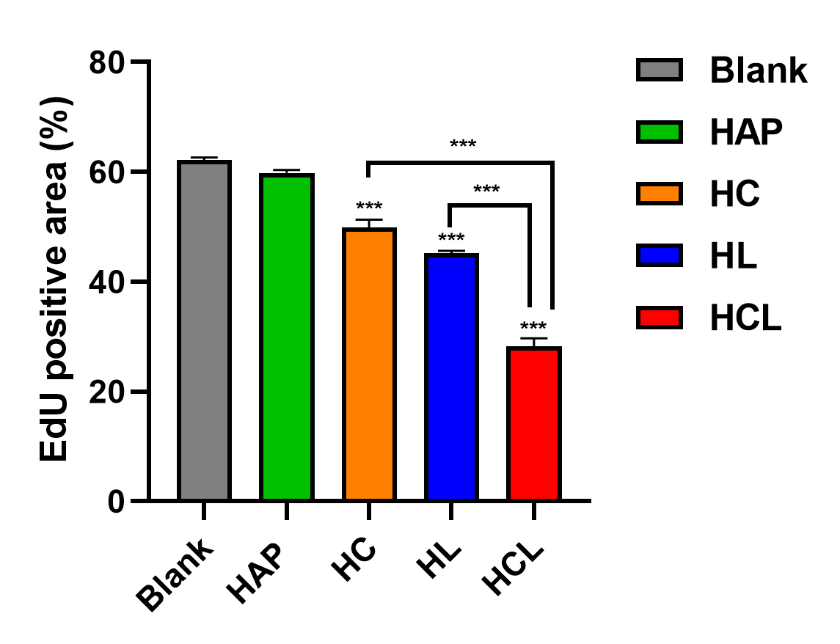


**Figure** **S3.** The statistics EdU-positive rates of CT26 cells after incubation with HAP, HC, HL and HCL NPs, **P* < 0.05, ***P* < 0.01, ****P* < 0.001.


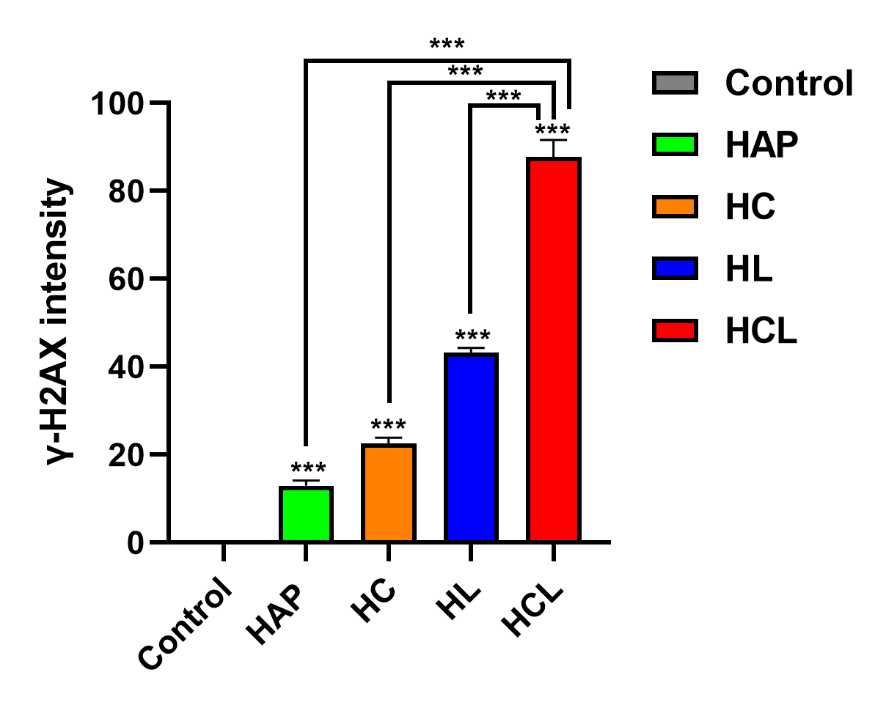


**Figure** **S4.** The statistical γ-H2AX fluorescence intensity in CT26 cells after incubation with HAP, HC, HL and HCL NPs, **P* < 0.05, ***P* < 0.01, ****P* < 0.001.


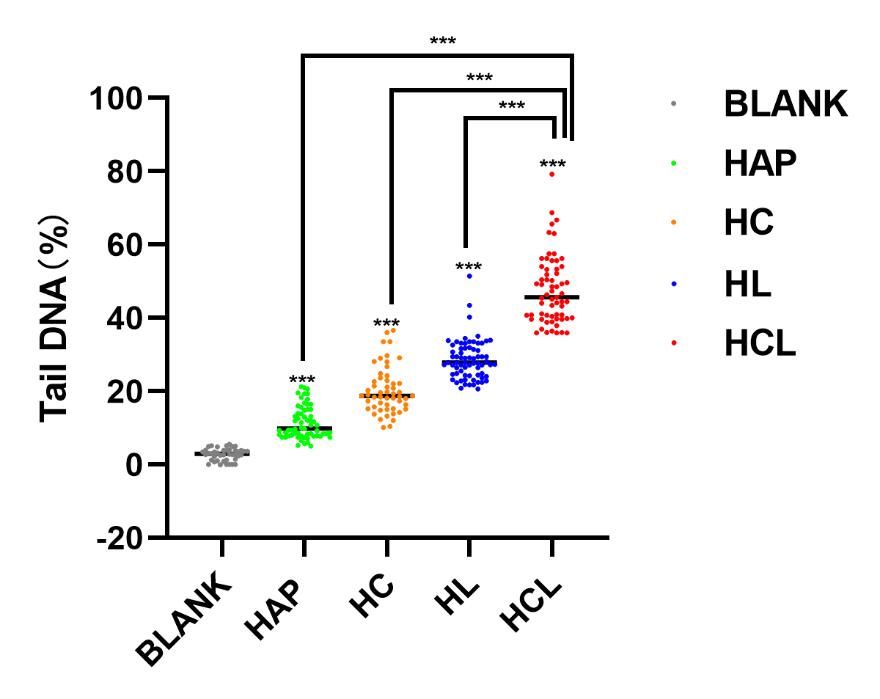


**Figure** **S5.** The statistical analysis of DNA damage accumulation was quantified by comet assay, **P* < 0.05, ***P* < 0.01, ****P* < 0.001.


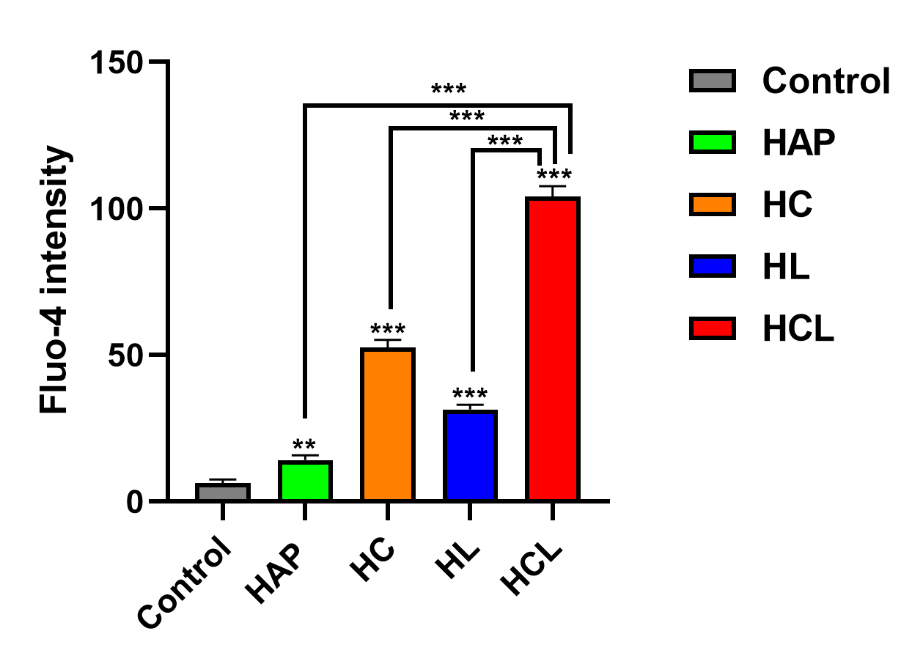


**Figure** **S6.** The statistical analysis of Fluo-4 fluorescence intensity in CT26 cells after incubation with HAP, HC, HL and HCL NPs, **P* < 0.05, ***P* < 0.01, ****P* < 0.001.


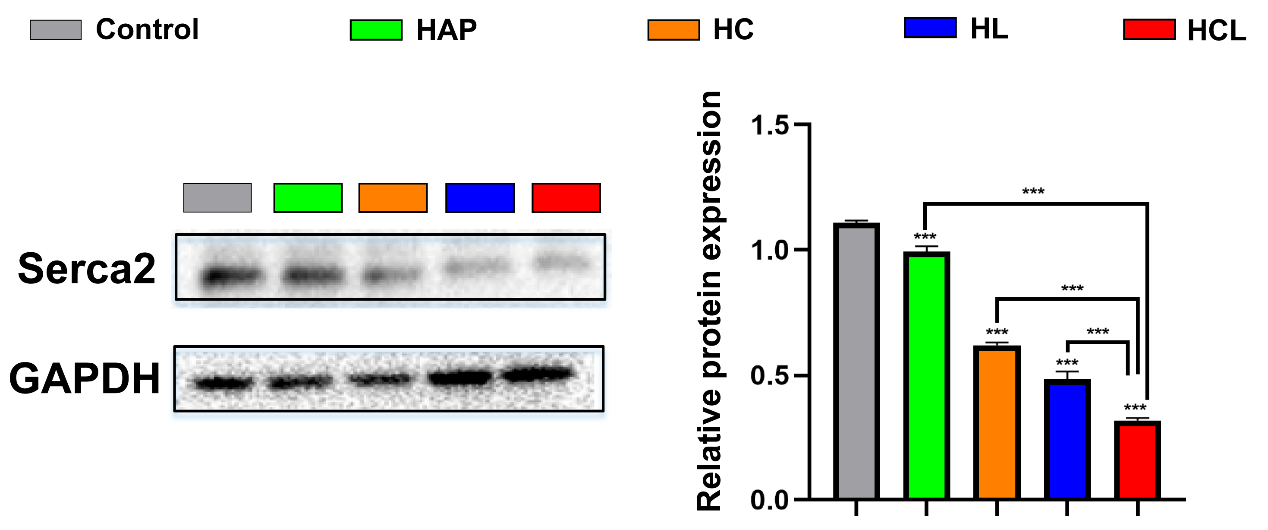


**Figure** **S7.** Western blot analysis of protein Serca2.


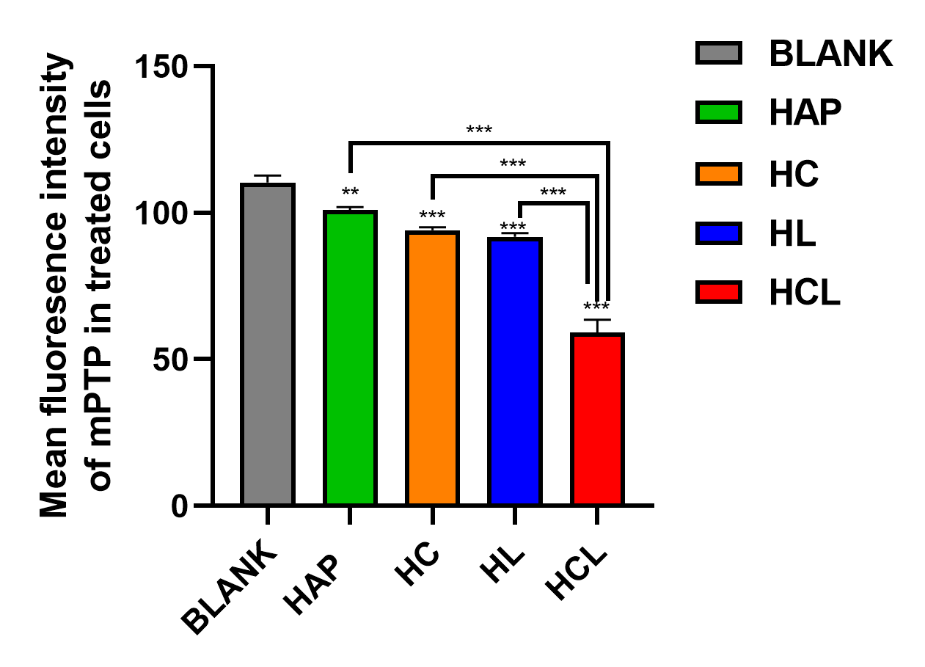


**Figure** **S8.** The statistical analysis of mPTP fluorescence intensity in CT26 cells after incubation with HAP, HC, HL and HCL NPs, **P* < 0.05, ***P* < 0.01, ****P* < 0.001.


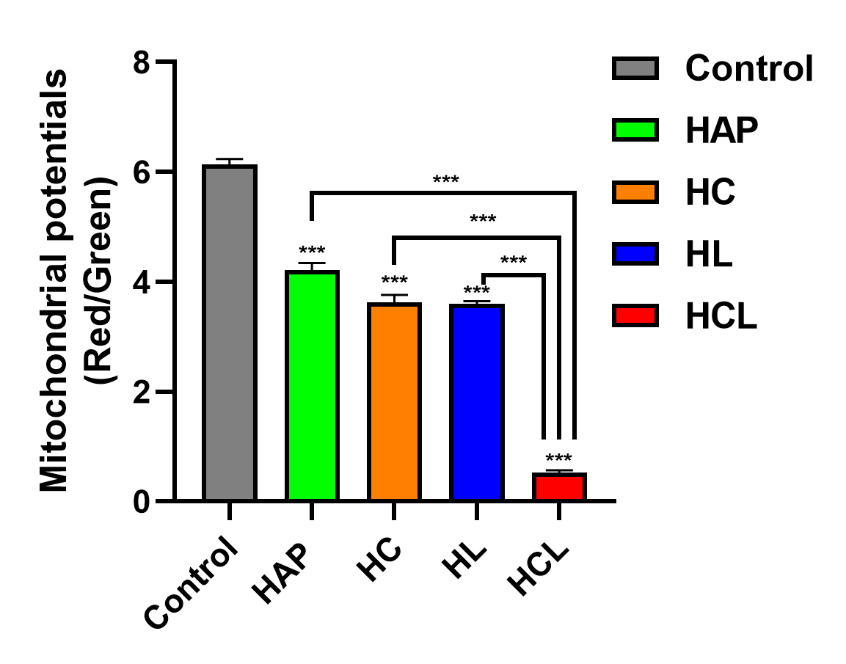


**Figure** **S9.** The statistical analysis of ratio of red/green fluorescence intensity in CT26 cells after incubation with HAP, HC, HL and HCL NPs, **P* < 0.05, ***P* < 0.01, ****P* < 0.001.


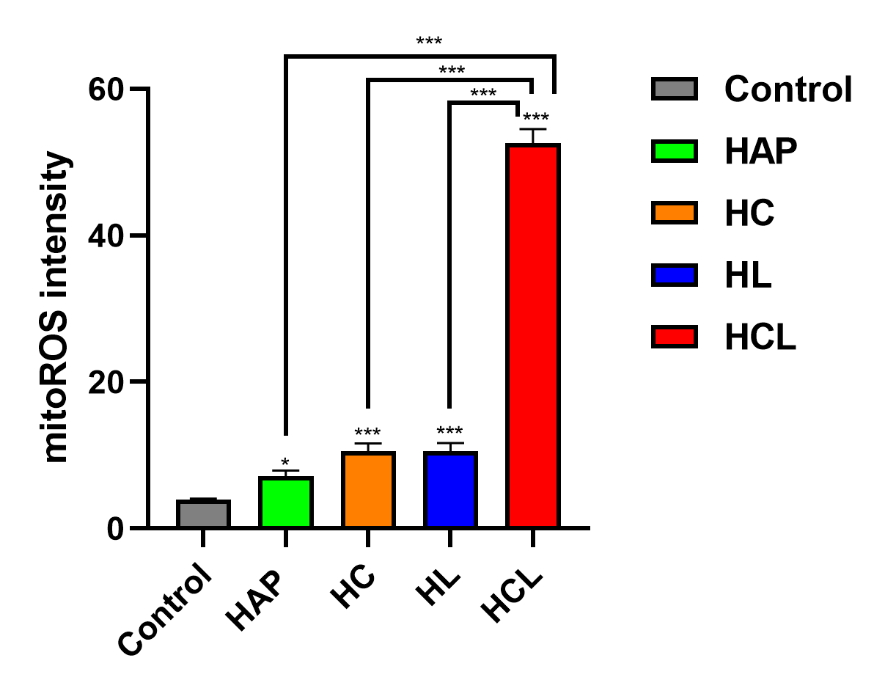


**Figure** **S10.** The statistical analysis of mitoROS fluorescence intensity in CT26 cells after incubation with HAP, HC, HL and HCL NPs, **P* < 0.05, ***P* < 0.01, ****P* < 0.001.


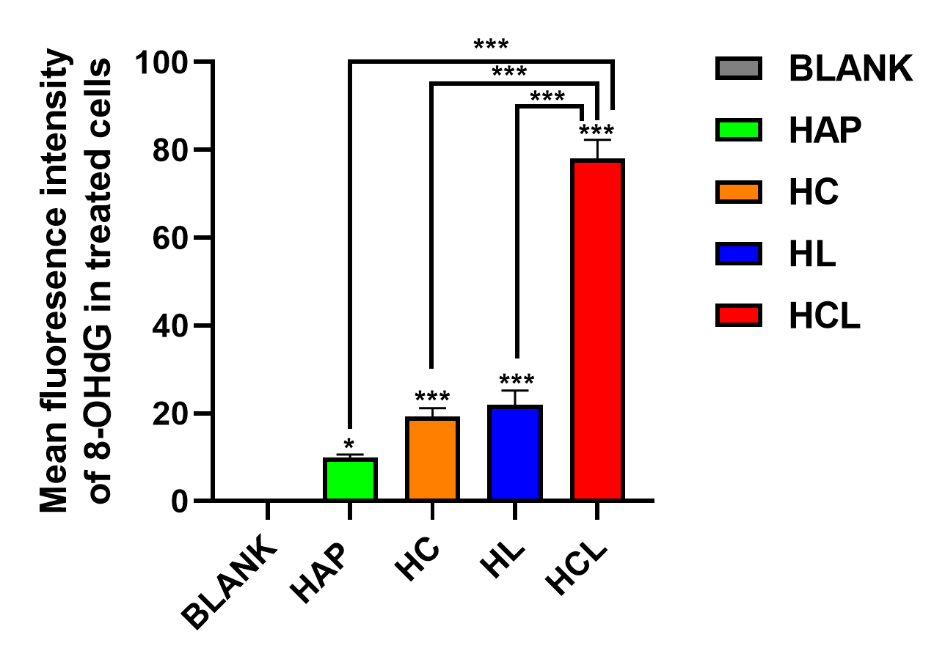


**Figure** **S11.** The statistical 8-OHdG fluorescence intensity in CT26 cells after incubation with HAP, HC, HL and HCL NPs, **P* < 0.05, ***P* < 0.01, ****P* < 0.001.


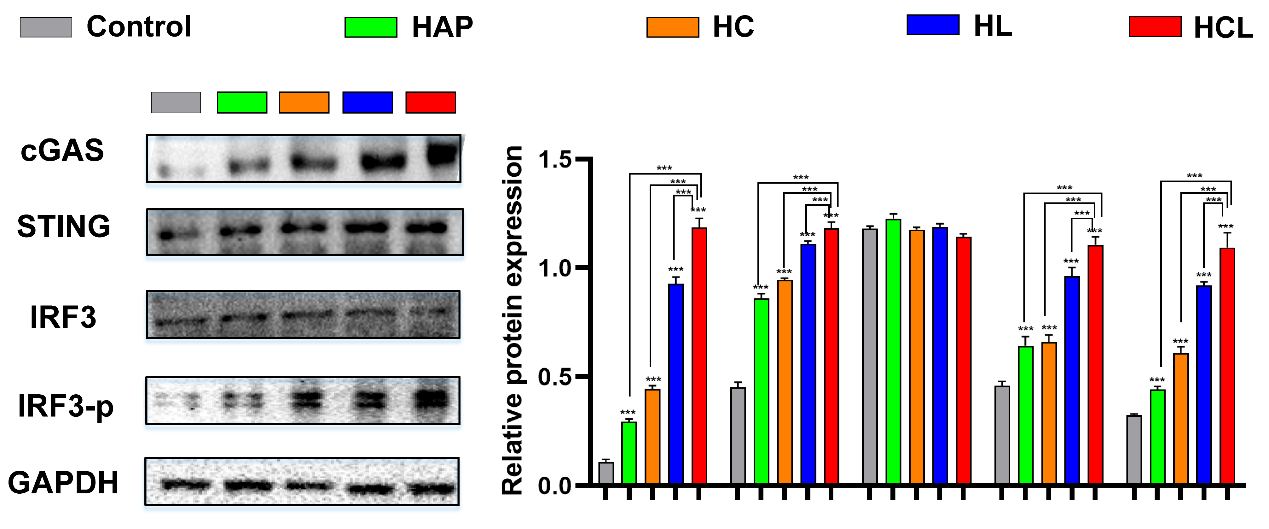


**Figure** **S12.** Western blot analysis of cGAS-STING pathway-related proteins in tumor tissues.


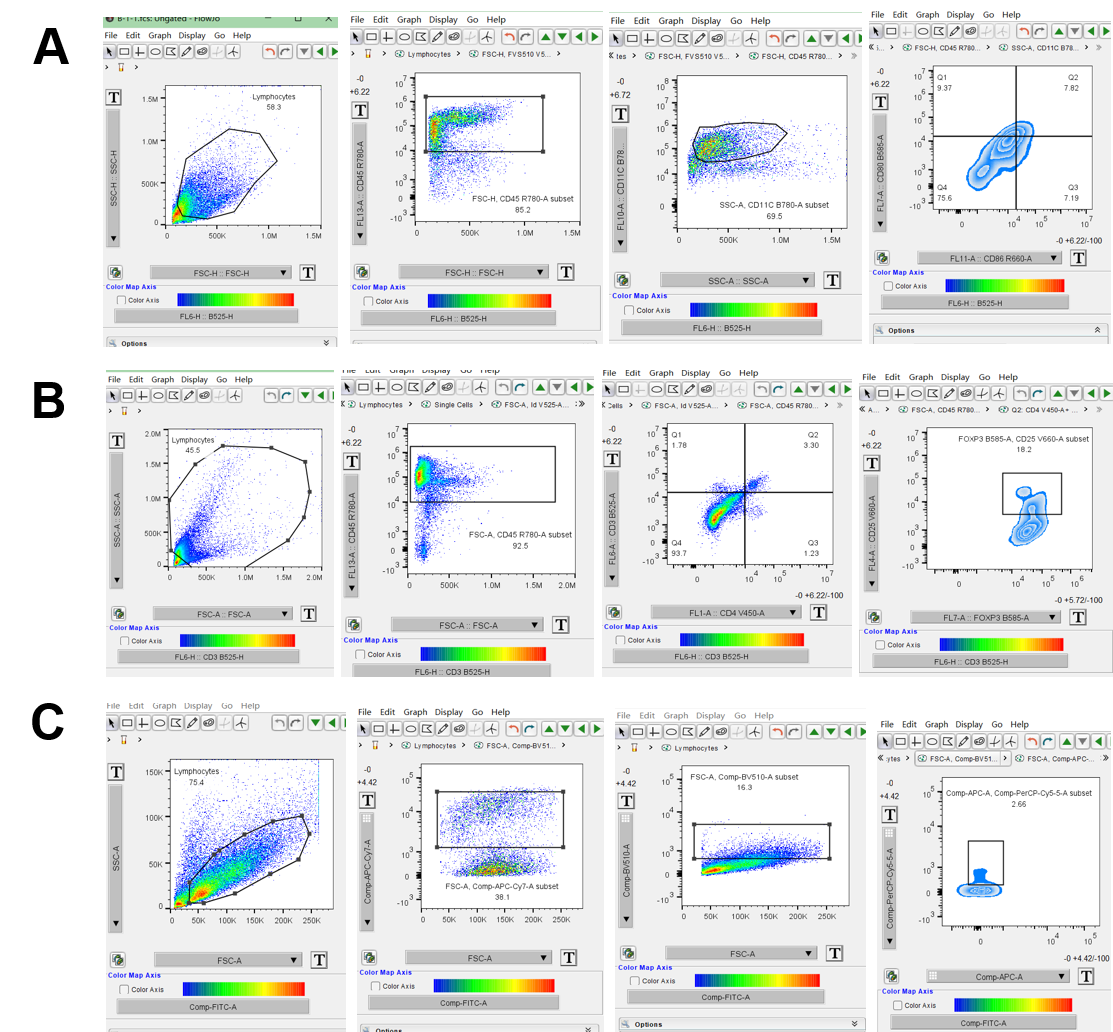


**Figure S13.** (A) The original data of flow cytometry results of the intratumor infiltration of DC cells (FSC, SSC and gate setting). (B) The original data of flow cytometry results of the intratumor infiltration of Treg cells (FSC, SSC and gate setting). (C) The original data of flow cytometry results of the intratumor infiltration of CD8+ T cells (FSC, SSC and gate setting).
